# Fluorescent Imprinted Nanoparticles for the Effective Monitoring of Irinotecan in Human Plasma

**DOI:** 10.3390/nano10091707

**Published:** 2020-08-29

**Authors:** Martina Tommasini, Elena Pellizzoni, Valentina Iacuzzi, Elena Marangon, Paola Posocco, Cristina Forzato, Paolo Bertoncin, Giuseppe Toffoli, Marina Resmini, Federico Berti

**Affiliations:** 1 Department of Chemical and Pharmaceutical Sciences, University of Trieste, Via Giorgieri 1, 34127 Trieste, Italy; martina.tommasini28@gmail.com (M.T.); pellizzoni.elena@gmail.com (E.P.); cforzato@units.it (C.F.); 2 PhD School in Nanotechnology, University of Trieste, Via Giorgieri 1, 34127 Trieste, Italy; valentina.iacuzzi@cro.it; 3 Department of Life Sciences, University of Trieste, Via Giorgieri 5, 34127 Trieste, Italy; pbertoncin@units.it; 4 CRO–National Cancer Institute, SOC–Experimental and Clinical Pharmacology, Via Gallini 2, 33081 Aviano (PN), Italy; elenamarangon@hotmail.com; 5 Department of Engineering and Architecture, University of Trieste, Via Valerio 6/1, 34127 Trieste, Italy; paola.posocco@dia.units.it; 6 School of Biological and Chemical Sciences, Queen Mary University of London, Mile End Road, London E14NS, UK

**Keywords:** imprinted nanogels, therapeutic drug monitoring, irinotecan, human plasma, fluorescence

## Abstract

Fluorescent, imprinted nanosized polymers for the detection of irinotecan have been synthesised using a napthalimide polymerisable derivative (2-allyl-6-[2-(aminoethyl)-amino] napthalimide) as functional monomer. The imprinted polymers contain ethylene glycol dimethacrylate (EGDMA) as a cross-linker and were prepared by high dilution radical polymerisation in dimethylsulphoxide (DMSO). The material was able to rebind irinotecan up to 18 nmol/mg with good specificity. Fluorescence emission at 525 nm (excitation at 448 nm) was quenched by increasing concentrations of irinotecan via a static mechanism and also in analytically useful environments as mixtures of human plasma and organic solvents. This allowed the direct detection of irinotecan (in the 10–30 μM range) in human plasma treated with acetonitrile; the limit of detection (LOD) was 9.4 nM, with within-run variability of 10% and day-to-day variability of 13%.

## 1. Introduction

**Figure 1:**
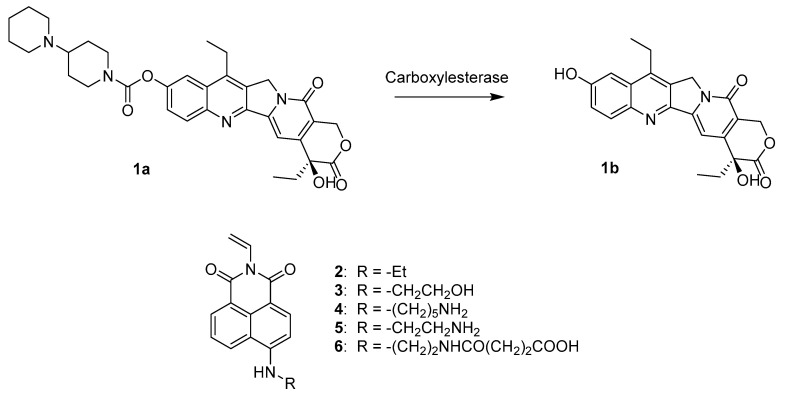
Structures of irinotecan (**1a**) SN-38 (**1b**) and of the functional monomers **2**–**6** used in this work.

Irinotecan 1a (Figure 1) [1], (Camptosar^®^, CPT11) (Figure 1) is an inhibitor of topoisomerase I largely used in the therapy of metastatic colorectal cancer. It is administered intravenously as the prodrug of its active metabolite SN-38 1b [2]. The molecule is administered as the hydrochloride salt, where the piperidine nitrogen, with a reported pKa of 8.7, is protonated [3].

The drug shows high pharmacokinetic variability and heavy secondary effects [4], and for this reason, its quantification in plasma is an essential step towards personalised therapy. Early pharmacokinetic studies reported an average plasma concentration of about 1 mg/L (170 nM) [5]. However, this initial reference value seems currently no longer reliable, and in our own clinical experience, we have also observed a range of concentrations between 17 nM and 17 μM with high variability [6]. Such variability highlights the urgent need for therapeutic drug monitoring (TDM) of this drug that can help in the clinical practice to identify the best dose regimen, therefore reducing side effects and resulting in a personalised treatment [7,8].

Irinotecan and SN-38 have been detected in human plasma by HPLC-UV, LC-MS and direct MALDI-MS [6,9,10,11]. Point-of-care devices for irinotecan monitoring have never been described, and they could be very helpful to optimise the therapy.

The key component of any sensing platform is the recognition element that allows it to specifically identify and bind the target analyte. Biomolecules such as antibodies and enzymes have been widely used for this purpose with a variety of targets; however, more recently, there has been a strong interest toward using polymers for recognition. Among the different approaches investigated, molecular imprinting has been shown to work effectively to deliver artificial biomimetic receptors with high molecular recognition properties [12].

Molecularly imprinted polymers (MIPs) are well-known in applications such as stationary phases and solid-phase extraction (SPE) for the detection of small molecules, including irinotecan [13], for coating of metal nanoparticles in surface-enhanced Raman spectroscopy (SERS) [14], and as drug delivery systems [15]. Applications in catalysis [16] and sensing [17] have been reported also with soluble micro- and nano-structured imprinted materials.

A MIP towards camptothecin was used in solid-phase extraction coupled with HPLC to develop an analytical methodology, with a good linearity in the range of 1–200 µg·mL^−1^ (2.9–574.2 µmol L^−1^) and a LOD of 0.13 µg mL^−1^ (0.4 µmol L^−1^) [18].

An imprinted polymeric material could be used as the sensing material for irinotecan and to generate a detectable signal upon interaction with the drug. Here, we present our work on the design, synthesis, and characterisation of fluorescent imprinted nanoparticles that were used for the detection and quantification of irinotecan in human plasma samples. This represents a first step towards the future development of a sensor for the rapid detection of irinotecan. The sensing nanomaterial exploits a fluorescent reporter directly embedded inside the matrix. Five fluorescent napthalimide derivatives (2–6) were evaluated by studying the interactions with the target analyte via ^1^H-NMR. The MIPs were characterised through dynamic light scattering (DLS) and transmission electron microscopy (TEM); rebinding of irinotecan was studied by HPLC and fluorescence measurements, first in simple environments and then in human plasma samples treated with acetonitrile.

## 2. Materials and Methods

### 2.1. Chemicals

Irinotecan hydrochloride was purchased by Bepharm ltd (Shangai, China). The other reagents and solvents were from Sigma-Aldrich (Milano, Italy). Human plasma (pool) was collected at CRO-National Cancer Institute of Aviano from 14 healthy volunteers—seven males and seven females in the 25–50 year age range. All subjects gave their informed consent for inclusion before they participated in the study. The study was conducted in accordance with the Declaration of Helsinki, and the protocol was approved by the Ethics Committee of CRO–National Cancer Institute (Project identification code 2009-25).

### 2.2. Instrumentation

NMR spectra (500 MHz) were recorded on a Varian 500 spectrometer (Palo Alto, CA, USA). HPLC analyses were run on an Agilent series 1100 liquid chromatograph (Santa Clara, CA, USA), with a Phenomenex (Torrance, CA, USA), Luna C18-5µ column with a column guard and a 20 µL loop. Fluorimetric titrations were performed on a CARY Eclipse (Varian) spectrometer with orthogonal geometry with a square cuvette of 0.5 cm optical path. TEM images were recorded with a Camera Olympus QUEMESA (Tokyo, Japan) and software RADIUS 2.0 (EMSIS) (Münster, Germany) on a TEM images Philips EM208 (Amsterdam, The Netherlands) at 100 kV using a 200-mesh copper grid with carbon film. Dynamic Laser Light Scattering was performed on a Zetasizer nano-S (Malvern Panalytical, Malvern, UK) instrument. Data analysis and regressions were carried out using Sigma Plot 13 (Sysdat Software Inc., San Jose, CA, USA).

### 2.3. ^1^H-NMR Titrations

Irinotecan HCl (2.3 mg, 3.40 µmol) was dissolved in 850 µL of DMSO-d_6_ to a 4 mM concentration, and 5 μL amounts of monomer mother solutions in DMSO-d_6_ were added to obtain final concentrations from 2 mM to 40 mM. ^1^H NMR spectra were recorded after each addition. The overall volume change upon additions was 60 μL (6.5%), and a control experiment was carried out by addition of the same volumes of solvent.

### 2.4. Synthesis of the Polymers (General Procedure)

The MIPs and their corresponding not imprinted polymers (NIPs) were prepared according to ref. [19]. The template and the functional monomer were stirred in anhydrous DMSO in a vial under an argon atmosphere, and then the cross-linker and AIBN were added. The vial was sealed and kept at 70 °C for four days. Each polymer was synthesised both in presence of the template molecule, and without it. The resulting clear solutions were dialysed (cut off 3.5 kDa) against water for two days, against a MeOH:AcOH (8:2) mixture for two days and against water for five additional days, changing the solvent twice a day.

The solutions were then freeze-dried to obtain fluffy solids. The amounts of reactants for the synthesis of each polymer are reported in Table 1, while % compositions of the reacting mixtures, yields and sizes are reported in Table 2.

### 2.5. Dynamic Light Scattering

0.25 mg mL^−1^ DMSO solutions of each material were filtered on 0.45 µm filters and the DLS measures were carried out in triplicate in a 1 mL quartz cuvette with light path of 1 cm.

### 2.6. Transmission Electron Microscopy

0.2 mg of MIP F were suspended in 1 mL of distilled water, and the mixture was stirred for 30 min. The resulting solution was diluted 40 times with distilled water and stirred for further 10 min. A drop was then placed on a grid coated with amorphous carbon and the solution was allowed to dry at room temperature; TEM images of MIP F were recorded.

### 2.7. Rebinding Tests

1.5 mg of each polymer were dissolved in 1.5 mL of a 100 μM 1a water solution. The mixtures were stirred at room temperature, and 200 μL aliquots were analysed by HPLC after centrifugation (10,000 rpm, 10 min) at different times (from 10 min to 24 h). The concentration of residual drug was obtained from a calibration curve (reported in the Supplementary Figure S1), and the amount captured by the polymer was calculated by difference. HPLC analyses were carried out at 25 °C with a 75:25 water:acetonitrile mobile phase (0.05% TFA), at a 1 mL/min flux and UV detection (363 nm).

### 2.8. Fluorimetry

The fluorescent MIPs/NIPs were dissolved in 3:1 acetonitrile:water mixtures at 60 μg/mL final concentration by diluting their mother 0.25 mg mL^−1^ DMSO solutions. Increasing amounts of **1a**, from 20 nM to 25 µM, were then added from mother DMSO solutions. Fluorescence emission spectra were recorded at 448 nm excitation. The spectra were recorded after 5 min from the addition of **1a**. Titration data were obtained at excitation and emission wavelengths of 448 and 525 nm. All the titrations were triplicated, and the emission was corrected for inner filter effects [20], using Equation (1):

(1) $$F_{corr}=F_{obs}\times 10^{\left(\frac{A_{exc}-A_{em}}{2}\right)}$$

where *F_corr_* is the corrected fluorescence emission, *F_obs_* is the observed one, and *A_exc_* and *A_em_* are the vis absorbance of **1a** at the excitation and emission wavelengths at the same concentration used in the titration point leading to *F_obs_*.

Stern–Volmer analyses were carried out according to Equation (2):

(2) $$\frac{F_{0}}{F}=1+K_{SV}\left[1a\right]\;\;\;\;\;\;\;\;where\;K_{SV}=k_{q}\tau_{0}$$

where *F_0_* is the initial value of fluorescence emission, *F* is the emission after the addition of 1a, *K_SV_* is the Stern–Volmer constant, *k_q_* is the quenching constant, and *τ_0_* is the decay time of the MIPs fluorophore, which was assumed equal to 8.3 ns as that of the naphtalimide derivatives reported in the literature [21].

### 2.9. Fluorescence Titrations in Plasma

Plasma samples were treated with three volumes of acetonitrile to precipitate proteins. The samples were submitted to centrifugation three times (10 min, 13,000 rpm, 4 °C). 400 µL of 60 µg/mL MIP F solution in a 3:1 acetonitrile:plasma mixture were then titrated with increasing concentrations of irinotecan from 5 nM to 2 µM, using excitation and emission wavelengths of 448 and 525 nm (5 nm slits) as described above. The titrations were carried out in triplicate, and the emission data were used to obtain a calibration curve as described in the result section.

## 3. Results and Discussion

### 3.1. Selection of Functional Monomers

**Figure 2:**
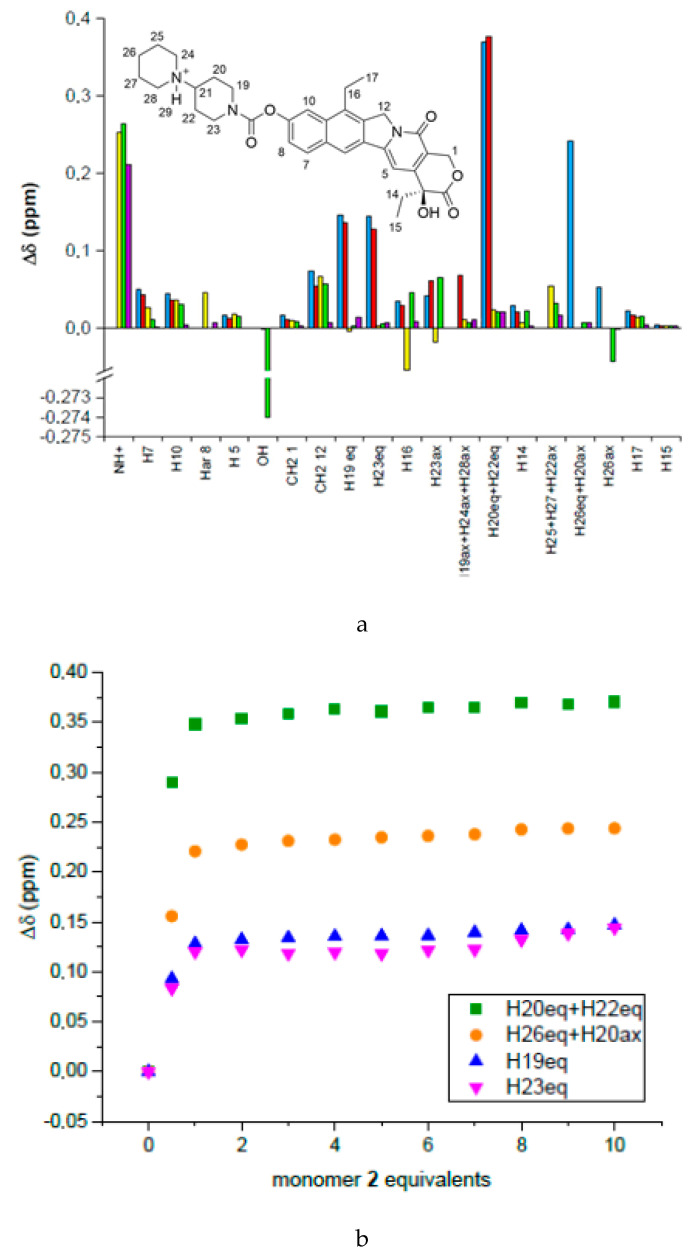
(**a**) Variation of chemical shifts of irinotecan protons after adding 10 equivalents of monomer **2** (yellow), **3** (cyan), **4** (blue), **5** (red), and **6** (green); (**b**) variation of chemical shift of selected irinotecan protons upon titration with monomer 5.

Irinotecan is a fluorescent molecule (its fluorescence emission spectrum is compared with that of naphtalimide derivative **5** in Supplementary Figure S2). Its fluorescence has been exploited for direct quantification from various biological matrices, as reported by different research groups [2,9,22,23]. Poujol et al. developed one of the most sensitive HPLC-fluorescence methods for irinotecan quantification in human plasma or saliva, with a LOD of 0.5 μg/L (about 0.7 nM) observed for both matrices [24]. However, its fluorescence intensity (360 nm excitation and 420 nm emission) can be altered by interferences with plasma components, and it cannot be directly used in a sensor for the direct quantification in plasma without a preliminary sample preparation step, for example, using liquid chromatography. According to our previous results on the development of imprinted nanogels towards sunitinib [19], we have first attempted to use polymerisable derivatives of amino acids as the potential fluorescent monomers also in this project, but their spectral properties were not suitable. We have therefore evaluated a number of different fluorophores known to emit in the region around 500 nm to avoid both plasma auto-fluorescence and the irinotecan fluorescence emission. We focused on naphtalimide derivatives **2**–**6**, able to emit around 520 nm when excited at 450 nm (Figure 1 and Figure S2) [25]. Such derivatives have been already employed as fluorescent MIP monomers [26,27,28]; for example, monomer **5** was employed by Rouhani et al. to develop a MIP sensor for caffeine, which showed a quenching of its fluorescence upon binding to the template with a limit of detection of 6.3 μM [29]. These very fluorescent molecules have been also used as probes for the detection of acidic drugs, biomolecules [30], and anions [31]. We have identified a small set of naphtalimide monomers (**2**–**6**) and synthesised them by literature methods [32,33,34,35]. They all contain the same N-allyl polymerisable group, and different chains substituted in the aniline ring, potentially capable to interact with irinotecan in different ways. The study of the binding interactions of the potential fluorophores with the target analyte was initially done by NMR [19]. The experiments were carried out in DMSO, the same solvent used during the polymerisation, to ensure that a strong interaction would remain in place. Proton NMR titrations of 4 mM irinotecan with increasing amounts of the synthesised fluorescent functional monomers (from 2 mM to 40 mM) were performed (Figure 2).

In general, following the addition of the monomers, most of irinotecan’s protons were shifted to higher fields, suggesting a shielding due to the close presence of the monomer (Figure 2a). Compounds **2** and **3** did not interact significantly with the drug (with the exception of the piperidinium proton), while derivatives **4, 5**, and **6** showed the largest proton shifts. In particular, compounds **4** and **5** showed a similar behaviour affecting mainly the protons of the first piperdidine ring (protons at C-20, C-22, C-19, C-23). (Figure 2) On increasing the amount of **4** and **5**, a plateau is obtained after the addition of one equivalent of functional monomer (Figure 2b). In reverse titrations carried out with monomers **4** and **5** at constant concentration and at increasing concentrations of irinotecan, large shifts of the aminoalkyl chains of the two monomers are observed (Supplementary Figure S3). The main interaction is therefore occurring between the amino groups of the partners, either by hydrogen bonding or proton exchange. Further π–π stacking interactions also occurred between the aromatic structure of napthalimide and the quinoline moiety of the anticancer agent, as suggested by changes in the shifts of the aromatic protons. A clear trend towards saturation of the change of chemical shift was not observed with the other monomers. The final decision was in favour of compound **5** rather than **4**; the conformational freedom of the two compounds, which is minor in the shorter chain of **5**, was also taken into account. Monomer **5** was thus used in all the preparations.

### 3.2. Synthesis and Characterisation of the Polymers

A small library of MIPs was synthesised using a combination of functional monomer **5** and the co-monomers methacrylic acid (MAA), acrylamide (AA), and N-isopropylacrylamide (NIPAM) while EGDMA was used as cross-linker, and the template used was in all cases of irinotecan. MAA was considered as the first option as irinotecan contains a tertiary amino group, and proton transfer was expected to lead to a strong ionic interaction. AA and NIPAM are both hydrogen donors and acceptors and could favourably interact with irinotecan in both ways. Moreover, the isopropyl group of NIPAM could be involved in hydrophobic interactions with aromatic systems of the target. Finally, the cross-linker EGMA is a hydrogen bond acceptor and could also interact with the hydroxyl groups present on irinotecan. For each preparation, a set of MIPs and NIPs were obtained. The characterisation of each polymer was based on three criteria: rebinding capacity towards irinotecan, specificity as measured by the imprinting factor, and finally ability to give a significant fluorescence change upon rebinding of the target. Nanogels were obtained via high dilution radical polymerisation, using a non-covalent approach for imprinting [36]. This methodology, developed by N. Graham, allows to obtain polymers with very low polydispersity, as previously demonstrated by our group [37] and others [38]. The choice of DMSO as the solvent allows good solubility of all the reacting monomers and cross-linker while allowing electrostatic interactions to take place during the imprinting stage. The critical monomer concentration was assumed at 1% (in weight), while the porogen solvent constituted the 99% (in weight). We have verified that irinotecan is stable under the conditions required by this kind of synthesis. The relevant data for all the MIPs are reported in Table 2. All polymer preparations were analysed by DLS and the size reported in Table 2 is the one obtained by number. With nanoparticles so small, often the data presented by intensity are negatively affected by even small contaminants and size by number is preferable [39,40]. DLS distributions for MIP and NIP F are reported in the Supplementary Materials.

**Figure 3:**
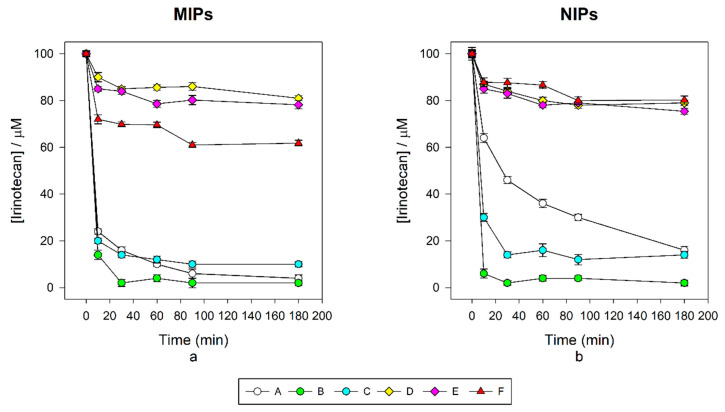
(**a**) Decay of irinotecan concentration in 100 μM solutions in the presence the MIPs; (**b**) decay of irinotecan concentration in 100 μM solutions in the presence of the NIPs.

A first set of MIPs was prepared using monomer **5** and MAA as co-monomer (MIPs A-C). MIPs A and B were obtained in high yields, together with their corresponding NIPs. The polymers showed high rebinding of irinotecan in water, capturing almost all the drug available already after 10 min (Figure 3). However, the corresponding NIPs were also able to capture significant amounts of irinotecan, and evidence of imprinting was observed only in MIP A, which gave a 1.6 imprinting factor. If the amount of MAA is reduced as in MIP C, both chemical yield and rebinding are reduced. MIPs A and B were unable to show any change in fluorescence emission upon titration with irinotecan, while some quenching was obtained by decreasing the amount of MAA as in C. The amount of quenching was rather small compared to the initial emission, but nevertheless, the Stern–Volmer and quenching constants, measured at 450 nm emission and 520 nm excitation after inner filter effects correction, were consistent with a static phenomenon.

As the unsatisfactory optical performance was likely due to preferential rebinding by MAA rather than by the fluorescent monomer, a second set of fluorescent imprinted polymers for irinotecan (MIPs D–E) was then prepared as follows: 15% (mol) of monomer **5**, and 15% (mol) of different commercially available co-monomers, as N-isopropyl acrylamide (NIPAM) and acrylamide (AA). Such materials were obtained in lower yield, and comparison of rebinding data confirms that MAA in MIP A-C was indeed responsible for most of the rebinding. Rebinding by MIPs D-E was by far lower, and unfortunately, the use of acrylamide or NIPAM improved neither MIPs’ specificity nor their fluorescence quenching. Our expectations about the co-monomers were thus frustrated by the experimental evidence: MAA is even too much able to interact with irinotecan and obscures any interaction with the fluorescent monomer, while AA and NIPAM were not helpful at all.

MIP F was therefore finally prepared, using 30% of **5**, without any co-monomer, and 70% of EGDMA as cross-linker. This material was obtained with a better yield compared to MIPs D-E, and its rebinding capacity was significantly higher (Figure 3). Moreover, the imprinting factor was enhanced to a more promising value of 2.5. In the synthesis of NIPs D, E, and F, the yield was clearly less favourable than that of the corresponding MIP. We have verified by UV-VIS that this is not an apparent effect due to residual captured irinotecan inside the MIPS. The effect is probably the result of a lack of a proximity catalytic effect by the template, which is not present in the NIPs, and becomes more evident as the monomers are here less reactive than MAA.

**Figure 4:**
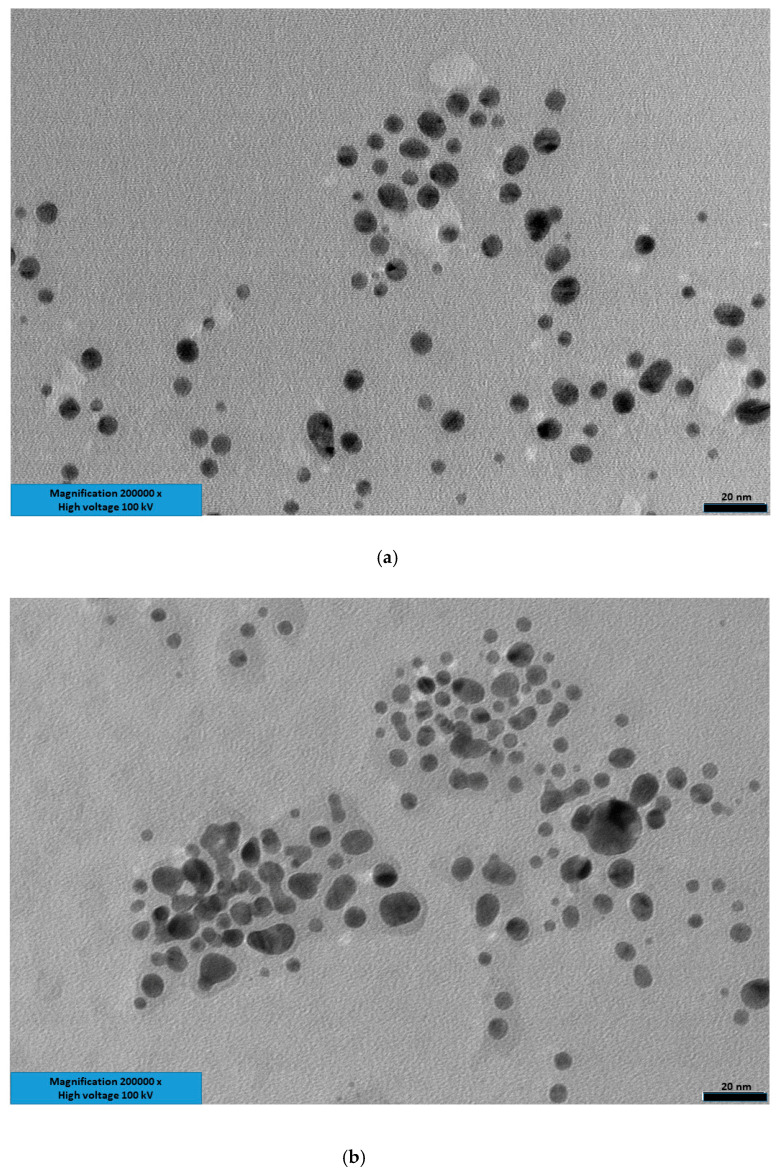
TEM images of MIP F; bar 20 nm. (**a**,**b**): two regions of the sample.

MIP F was also investigated by transmission electron microscopy using a carbon-coated grid. The relative images are reported in Figure 4 and gave an average particle size of 6.2 ± 3.4 nm. This result was consistent with the one obtained by DLS, as in TEM measures, the solvation sphere is obviously not present. The particles dimensions observed at TEM were comparable to the ones reported in the literature for similar MIPs. The higher polydispersity observed in TEM in comparison to DLS is most likely due to the formation of small aggregate fractions on drying the solvent on the TEM grid.

### 3.3. Fluorimetry and Evaluation in Human Plasma

**Figure 5:**
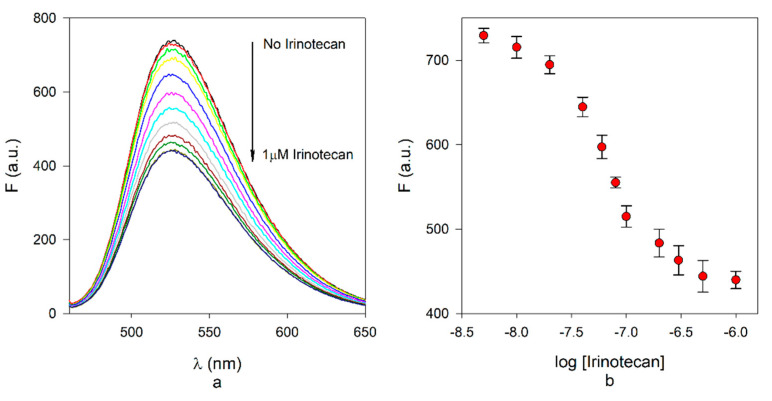
(**a**) Emission spectra of 60 μg/mL MIP F at increasing concentrations of irinotecan in 3:1 acetonitrile:water (excitation 448 nm); (**b**) fluorescence decrease at 525 nm of 60 μg/mL MIP F at increasing concentration of irinotecan in 3:1 acetonitrile:water.

The fluorescence studies were performed directly with MIP F in a solution of acetonitrile:water (3:1 *v/v*), as this medium was the same as the treated plasma samples. Optimisation of the fluorescence emission was carried out by varying the concentration of polymer used. A concentration of 60 μg/mL MIP was identified as the best one, showing a sufficiently high signal that would allow a clean and reproducible change of emission. The fluorescence emission spectra were recorded after five minutes following the addition of 1a (Figure 5a), as this was sufficient to stabilise the signal after the addition. The decay of the maximum wavelength emission is reported in Figure 5b.

The emission data are reported in Figure 5 as the fluorescence read from the instruments; this allows to see clearly that quenching is not complete, and involves about 38% of the initial emission, reaching a plateau at 1 μM irinotecan. Although not complete, the decrease of emission is large enough to allow its evaluation, and a Stern–Volmer analysis was carried out first (Supplementary Figure S4). The trend is linear in the 5–100 nM range and gave a Stern–Volmer constant of 4.35 × 10^6^ L mol^−1^; assuming a decay time of the fluorophore of 8.3 ns [21], a quenching constant of 5.24 × 10^14^ L mol^−1^ s^−1^ was obtained, fully consistent with static quenching. We have carried out some attempt to improve the amount of quenching, and actually, at concentrations of irinotecan exceeding 3 μM, and up to 25 μM, a further 15% amount of quenching can be obtained (Supplementary Figure S5). This change could be either due to a significant dynamic quenching at high irinotecan concentrations or to the existence of a further population of low affinity-low specificity binding sites in the MIP. Nevertheless, we have not considered presently this second decrease of emission as useful in the evaluation of the dynamic range of the MIP due to the occurrence of the plateau at 1 μM. This limit could not be overcome by changing the experimental conditions, and the residual emission of the MIP is most likely due to a fraction of fluorogenic monomers that is buried inside the polymeric nanoparticles and cannot interact with irinotecan. Attempts to increase the polymer concentration up to 120 μg/mL resulted in aggregation and precipitation of the sample.

**Figure 6:**
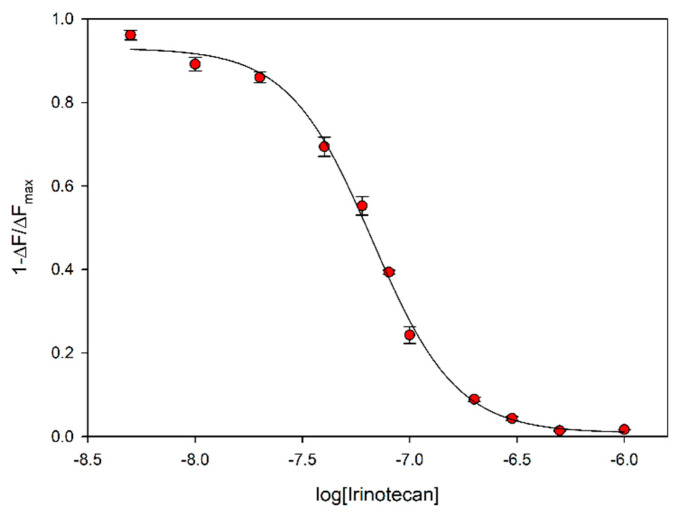
Fluorescence decrease of 60 μg/mL MIP F at increasing concentration of irinotecan in 3:1 acetonitrile:human plasma. The decrease is reported as the fraction of the maximum one.

As MIP F showed suitable characteristics as potential recognition element for a sensing device, in the low concentration range, we decided to further explore its properties with human plasma. Given that, in our clinical practice, plasma samples are currently treated with acetonitrile to denature proteins prior to LC-MS analysis, we decided to use the same mixture of acetonitrile and plasma [6]. On switching from 3:1 acetonitrile:water mixtures to 3:1 acetonitrile:plasma, no significant loss of sensitivity was observed, and the titration curves of fluorescence decay were almost superimposable to those obtained in the first measures in water. The plot is reported in Figure 6, where emission is given in a relative way, to allow evaluation of the curve as a potential calibration curve for the detection of irinotecan. Normalisation has been carried out by taking the maximum change in emission as the difference between the initial emission and the emission read at 1 μM irinotecan. The normalised curve is presented here, as in principle, it can be replicated on moving to other instruments.

In this experiment, a high sensitivity for irinotecan was observed, with a LOD of 12 nM, evaluated with the 3σ method. The LOD in water was found to be 16 nM, closely similar to that in plasma. The intra-assay precision in plasma:acetonitrile on a triplicated titration was 10.2% in the whole dynamic range. The MIP sensor gives a very stable and reproducible response during time, and the experiments were carried out over one month. The sensitivity was unchanged along this time time, with an average LOD of 10 nM. The inter-assay precision of the whole curve was found to be 13.2%.

The dynamic range investigated covers the low concentration side of the therapeutic range of irinotecan, which was found from 17 nM to 17 μM in our collection of clinical samples. In order to evaluate the potential of a detection system exploiting MIP F for irinotecan quantification in human plasma, we have obtained a calibration curve from a fit of the fluorescence to a tetraparametric logistic curve (Equation (3)).

(3) $$1-\frac{\Delta F}{\Delta F_{max}}=d+\frac{a-d}{1+10^{\left(\left[1a\right]logcb\right)}}$$

**Figure 7:**
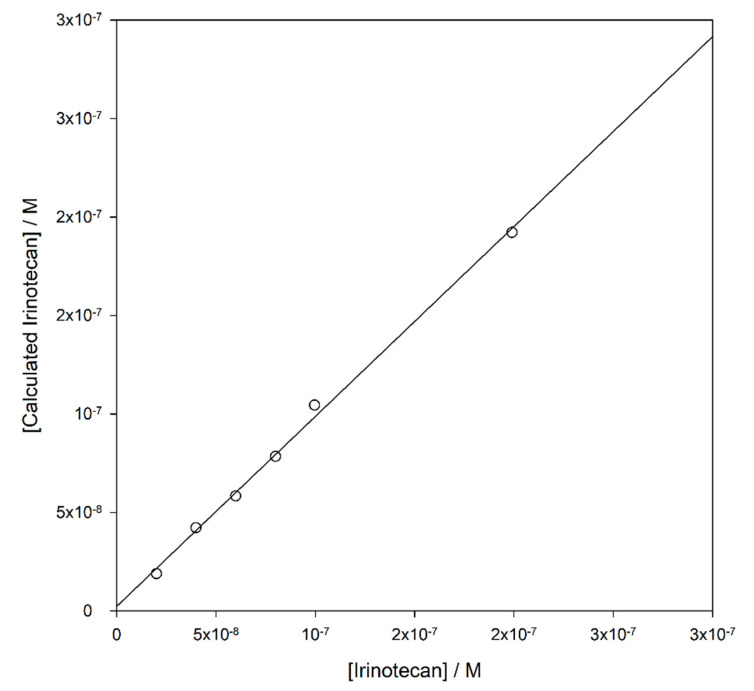
Correlation between the concentration of irinotecan in plasma samples and the calculated values from Equation (3).

The fit gave *r* > 0.9985 and *r*^2^ > 0.9970, with standard error on estimates 0.0247 an *p* < 0.0001 on the best-fitted parameters a–d. Using Equation (3) and its parameters, a very satisfactory outcome is obtained when the concentration of irinotecan is calculated in a series of samples obtained by dissolving irinotecan in the same pool of human plasma used to build the calibration curve, and the correlation between real and calculated values is reported in Figure 7.

The data are linearly correlated with *r*^2^ = 0.9955, a slope of 1.0001 and an intercept of 2.35 nM. The average accuracy of the calculated data is −0.6%. Although less challenging than a measure of recovery from authentic patient samples of known concentration before and after spiking (such samples are presently not available to us), this test shows nevertheless that the fluorescence data are very stable.

As to the selectivity of the system, a first indication is given by the fact that, on moving from solvent to human plasma, no changes in the performance are observed. The titration curve in plasma is almost superimposable to that obtained in solvent, and this means that no interfering compounds are present in plasma. As to other drugs that are usually co-administered with irinotecan, the only one in our experience is paracetamol, which does not affect the emission of the imprinted nanoparticles when preliminarily tested at 1 μM.

## 4. Conclusions

In conclusion, we have prepared a set of MIPs that can rebind irinotecan with good sensitivity. The imprinted fluorescent nanomaterial can be prepared and purified in cheap way and in less than one week. The fluorescence change of the napthalimide-containing MIP upon rebinding of the analyte has allowed the detection of irinotecan in human plasma, at nanomolar levels, after simple treatment of plasma with acetonitrile and removal of the proteins. The proof of concept given is encouraging towards the development of a portable device with sensors based on fluorescent imprinted nanoparticles for the detection of irinotecan. The present format of the system is clearly not technologically developed up to the level required for a self-analysis carried out by the patient, and several steps are still required. The main one is the immobilisation of the sensing material on a solid support, coupled with a portable, small, fluorimeter and a flow system to prepare the sample. We are currently working on these points; however, we have shown here that an imprinted nanomaterial able to change its optical properties on recognising irinotecan can be conveniently obtained in a simple way.

## Figures and Tables

**Table 1:** Composition of the polymerisation mixtures.

Polymer	Template [mg]	Functional Monomer [mg]	AIBN [mg]	Co-Monomer [mg]	Cross-Linker [mg]	DMSO [mL]
MIP-A	41.0	10.0	17.0	MAA 6.1	46.2	6.61
NIP-A	-					
MIP-B	41.0	15.0	17.0	MAA 12.2	59.5	7.88
NIP-B	-					
MIP-C	41.0	15.0	17.0	MAA 4.3	46.0	5.92
NIP-C	-					
MIP-D	41.0	15.0	17.0	NIPAM 5.7	46.2	6.06
NIP-D	-					
MIP-E	41.0	15.0	17.0	AA 3.5	46.2	5.87
NIP-E	-					
MIP-F	41.0	30.0	17.0	-	46.2	6.85
NIP-F	-					

**Table 2:** Characterisation of the imprinted materials.

MIP	Monomer 5 % ^a^	Co-Monomer% ^a^	Cross-Linker % ^a^	YieldMIP (NIP)% ^b^	Irinotecan Rebinding ^c^ nmol/mg	Specificity IF ^d^	Fluorimetry % quenching ^e^ *K_SV_* ^f^ 10^5^ L/mol	DLS Size(nm) ^g^ PDI ^h^
A	10	MAA	70	92	44	1.6	-	16.1 ± 1.0
		20		(98)				0.062
B	10	MAA	60	93	49	1.0	-	40.9 ± 2.3
		30		(80)				0.056
C	15	MAA	70	58	25	0.6	17	19.0 ± 6.1
		(15)		(63)			0.101	0.136
D	15	NIPAM (15)	70	69	8	0.9	-	15.7 ± 1.4
				(49)				0.089
E	15	AA	70	73	5	0.7	-	27.9 ± 2.6
		15		(41)				0.093
F	30	-	70	77	19	2.5	38	13.1 ± 2.0
				(42)			43.5 ± 1.8	0.153

^a^: mol% composition of the reacting mixtures leading to MIPs (% amount referred to the reactive monomers, excluding the template); ^b^: % yield calculated from the total mass of reagents and the mass of the resulting MIPs (and NIPs); ^c^: measured by HPLC after 20 min; ^d^: ratio between the amount of irinotecan captured by the MIP and that captured by its corresponding NIP; ^e^: % amount of quenching of the emission of 60 μg/mL MIPs in 3:1 mixtures of methanol and water (MIPs A-E) or acetonitrile and water (F) after the addition of 1 μM irinotecan; ^f^: from Stern–Volmer plots in the 10 nM–10 μM range of irinotecan added; ^g^: size distribution by number; ^h^: polydispersity index as the ratio between the standard deviation of particles size and their average size.

## Supplementary Materials

The following are available online at https://www.mdpi.com/2079-4991/10/9/1707/s1, Figure S1: HPLC calibration curve for irinotecan, used to measure residual irinotecan concentrations in rebinding tests. Slope 38.9204, intc. 68.5995, *r*^2^ 0.9984, Figure S2: Fluorescence emission spectra of 250 nM irinotecan and of 1 M naphtlimide 5, Figure S3: Change of chemical shifts of protons 13 (compound **5**) and 16 (compound **6**) upon titration with irinotecan, Figure S4: Stern–Volmer plot for the fluorescence titration of MIP F with irinotecan, Figure S5: Quenching of the fluorescence emission of MIP F in the high irinotecan concentration range (3–25 M), Figure S6: Size distribution by number of MIP F, Figure S7: Size distribution by volume of MIP F, Figure S8: Size distribution by intensity of MIP F, Figure S9: Size distribution by number of NIP F, Figure S10: Size distribution by volume of NIP F, Figure S11: Size distribution by intensity of NIP F.
