# Supplementary material for: Fluorescent Imprinted Nanoparticles for the Effective Monitoring of Irinotecan in Human Plasma
Source: Nanomaterials (Basel). 2020 Aug 29;10(9):1707. doi: 10.3390/nano10091707 (PMC7558923; doi:10.3390/nano10091707)
Supplement: Supplementary file 1 [file nanomaterials-10-01707-s001.pdf]

nanomaterials

Article

Fluorescent Imprinted Nanoparticles for the Effective Monitoring of Irinotecan in Human Plasma

Martina Tommasini [1], Elena Pellizzoni [1,2], Valentina Iacuzzi [2,3], Elena Marangon [4], Paola Posocco [5], Cristina Forzato [1], Paolo Bertoncin [3], Giuseppe Toffoli [4,*], Marina Resmini [6,*] and Federico Berti [1,*]

1 Department of Chemical and Pharmaceutical Sciences, University of Trieste, Via Giorgieri 1, 34127 Trieste, Italy; martina.tommasini28@gmail.com (M.T.); pellizzoni.elena@gmail.com (E.P.); cforzato@units.it (C.F.)
2 PhD School in Nanotechnology, University of Trieste, Via Giorgieri 1, 34127 Trieste, Italy; valentina.iacuzzi@cro.it
3 Department of Life Sciences, University of Trieste, Via Giorgieri 5, 34127 Trieste, Italy; pbertoncin@units.it
4 CRO–National Cancer Institute, SOC–Experimental and Clinical Pharmacology, Via Gallini 2, 33081 Aviano (PN), Italy; elenamarangon@hotmail.com
5 Department of Engineering and Architecture, University of Trieste, Via Valerio 6/1, 34127 Trieste, Italy; paola.posocco@dia.units.it
6 School of Biological and Chemical Sciences, Queen Mary University of London, Mile End Road, London E14NS, UK
* Correspondence: gtoffoli@cro.it (G.T.); m.resmini@qmul.ac.uk (M.R.); fberti@units.it (F.B.); Tel.: +39-040-558-3921 (F.B.)

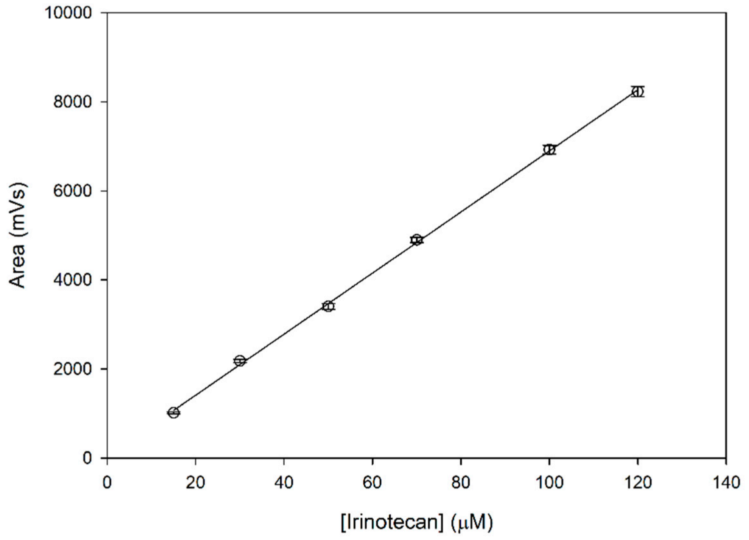

Figure S1. HPLC calibration curve for irinotecan, used to measure residual irinotecan concentrations in rebinding tests. Slope 38.9204, intc. 68.5995, r^2 0.9984.

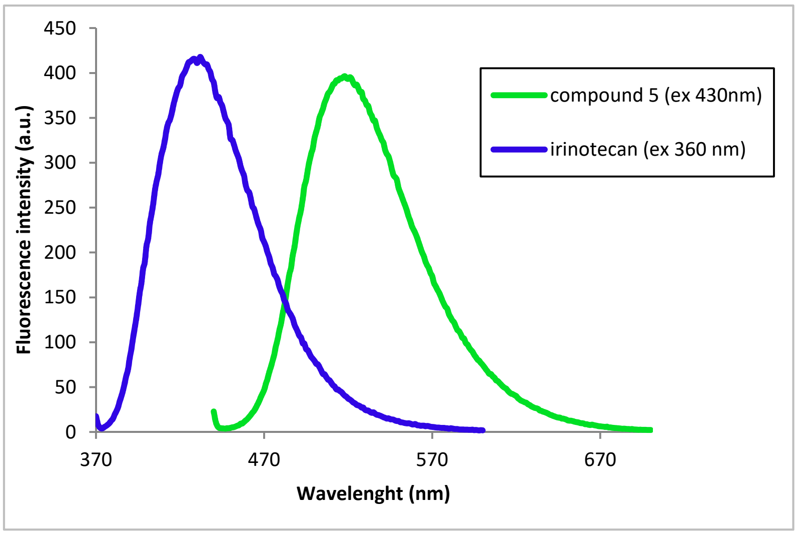

Figure S2. fluorescence emission spectra of 250 nM irinotecan and of 1 µM naphtlimide 5.

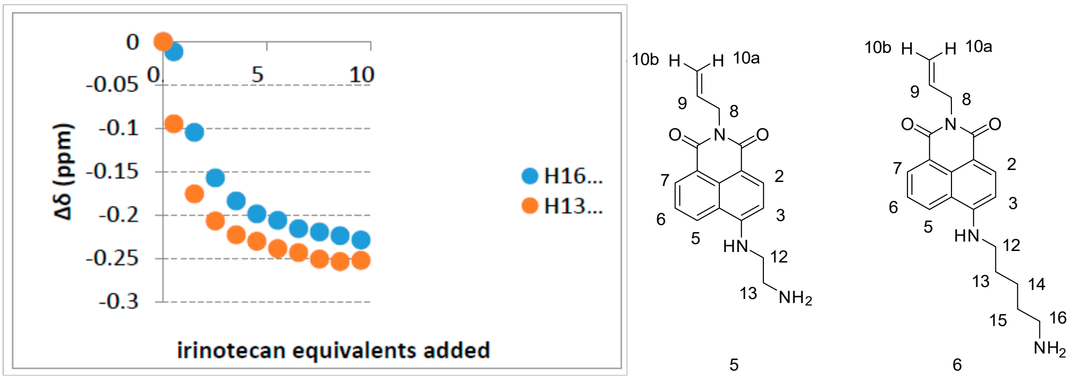

Figure S3. change of chemical shifts of protons 13 (compound **5**) and 16 (compound **6**) upon titration with irinotecan.

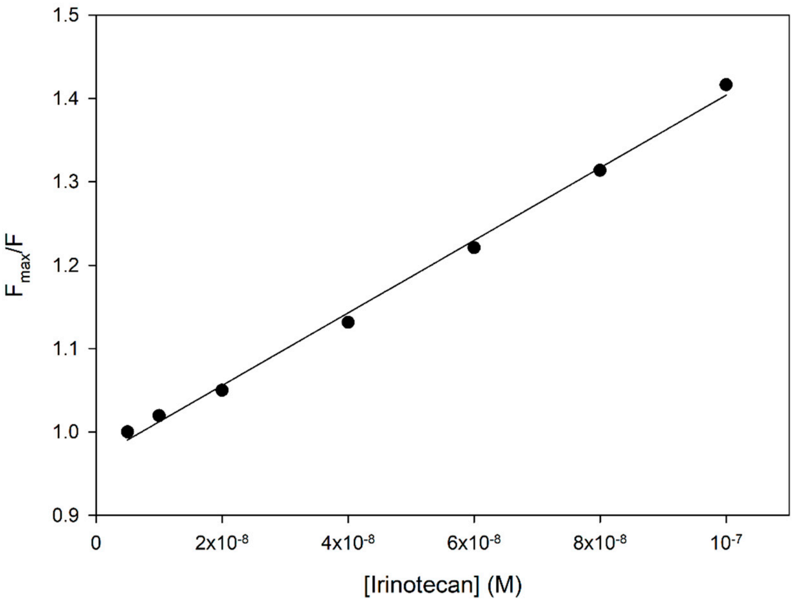

Figure S4. Stern-Volmer plot for the fluorescence titration of MIP F with irinotecan.

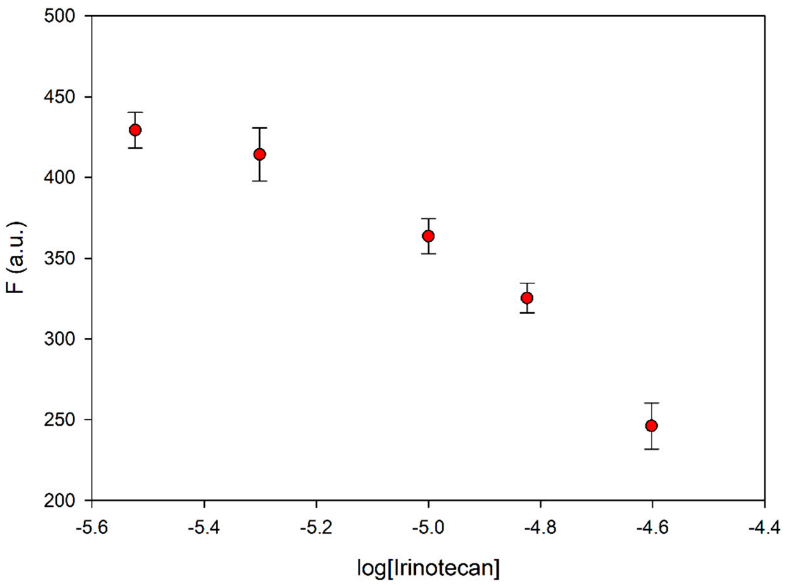

Figure S5. quenching of the fluorescence emission of MIP F in the high irinotecan concentration range (3–25 μM).

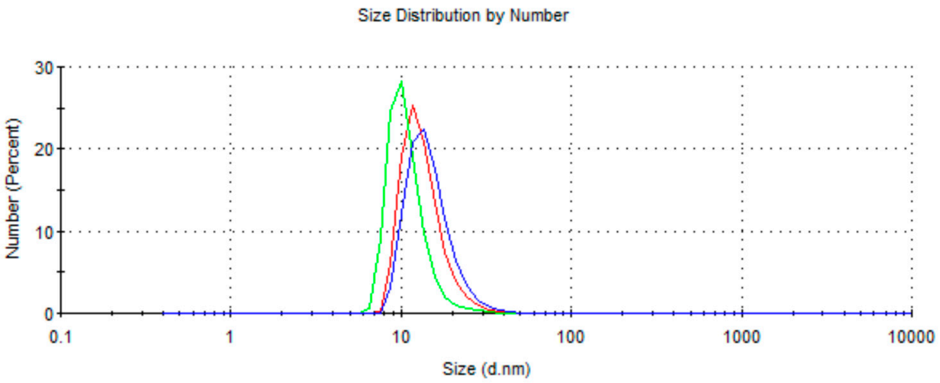

Figure S6. size distribution by number of MIP F.

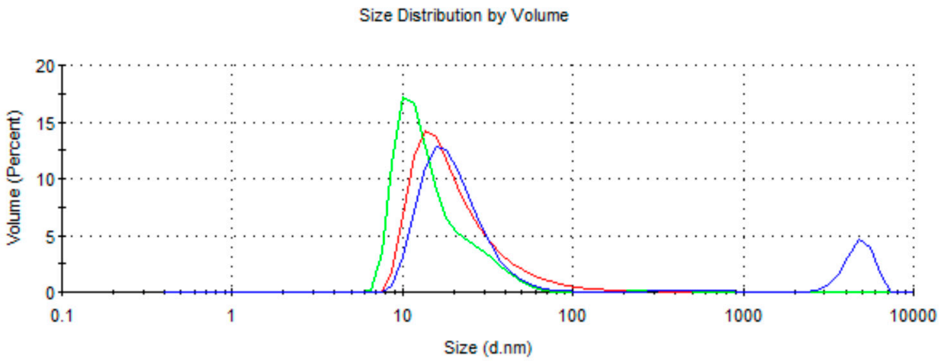

Figure S7. size distribution by volume of MIP F.

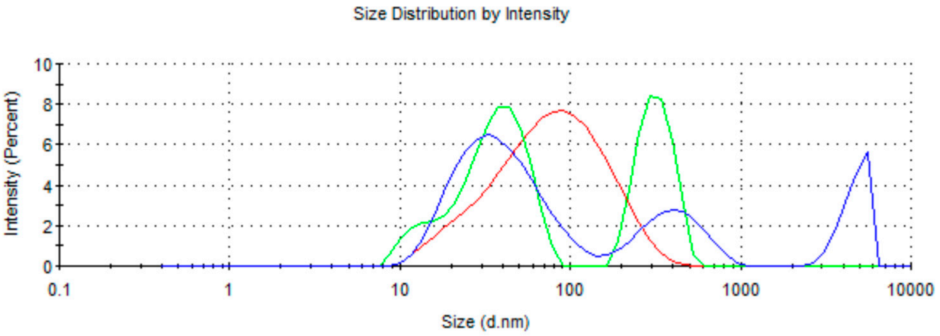

Figure S8. size distribution by intensity of MIP F.

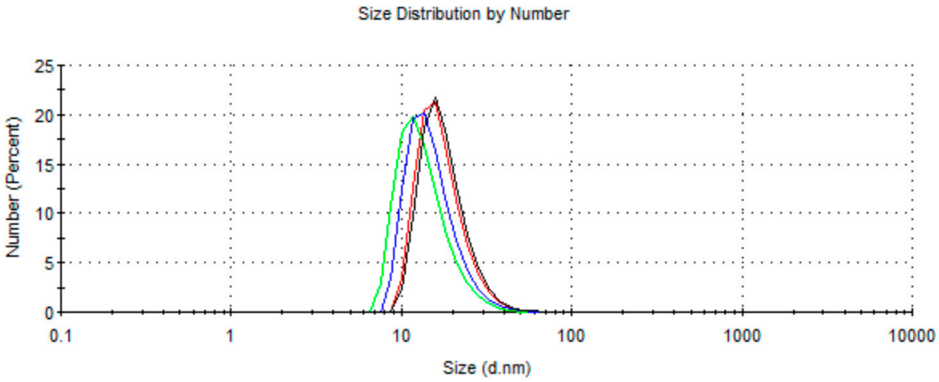

Figure S9. size distribution by number of NIP F.

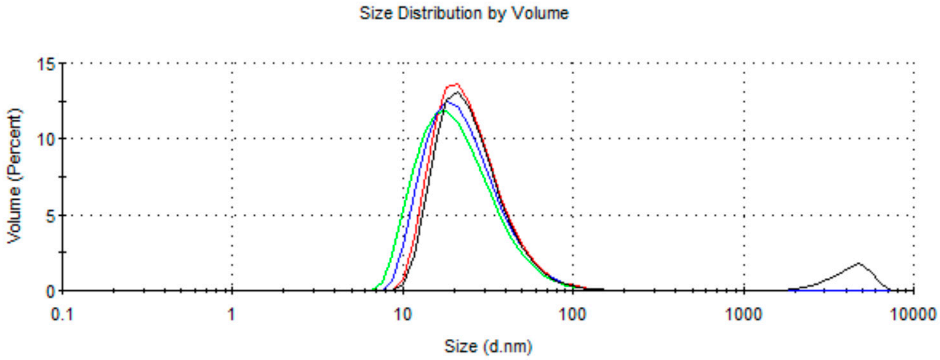

Figure S10. size distribution by volume of NIP F.

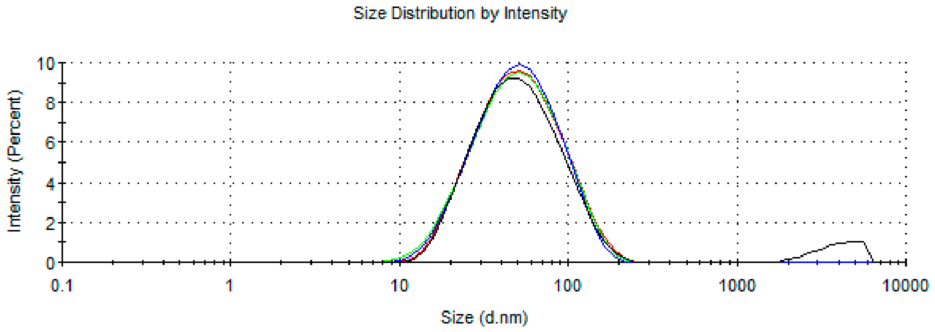

Figure S11. size distribution by intensity of NIP F.

© 2020 by the authors. Licensee MDPI, Basel, Switzerland. This article is an open access article distributed under the terms and conditions of the Creative Commons Attribution (CC BY) license (http://creativecommons.org/licenses/by/4.0/).
